# Identification of Nutritional Ingredients and Medicinal Components of *Pueraria lobata* and Its Varieties Using UPLC-MS/MS-Based Metabolomics

**DOI:** 10.3390/molecules26216587

**Published:** 2021-10-30

**Authors:** Xiaohong Shang, Ding Huang, Ying Wang, Liang Xiao, Ruhong Ming, Wendan Zeng, Sheng Cao, Liuying Lu, Zhengdan Wu, Huabing Yan

**Affiliations:** 1 Cash Crops Research Institute, Guangxi Academy of Agricultural Sciences, Nanning 530007, China; shxh789@gxaas.net (X.S.); wangying1983@gxaas.net (Y.W.); xiaoliang0420@gxaas.net (L.X.); zengwendan@gxaas.net (W.Z.); caosheng@gxaas.net (S.C.); luliuying@gxaas.net (L.L.); wuzhengdan@gxaas.net (Z.W.); 2 College of Pharmacy, Guangxi University of Chinese Medicine, Nanning 530200, China; huangd@gxtcmu.edu.cn (D.H.); mingrh@gxtcmu.edu.cn (R.M.)

**Keywords:** *Pueraria lobata*, UPLC-MS/MS, metabolite profiling, primary metabolites, secondary metabolites

## Abstract

*Pueraria lobata* and its variety *P. lobata* var. thomsonii are both traditional Chinese medicines that have high nutritional and medical value; whereas another variety, *P*. *lobata* var. montana has low nutritional and medicinal value and can cause ecological disasters. The material basis of different nutritional and medicinal values, which are caused by metabolite differences among these varieties, remains to be further clarified. Here, we performed ultra performance liquid chromatography-tandem mass spectrometry based widely targeted metabolome analysis on *Pueraria lobata*, *P. lobata* var. thomsonii, and *P*. *lobata* var. montana. Among them, a total of 614 metabolites were identified, and distinguished from each other using orthogonal partial least squares discriminant analysis. Our results suggest that the nutritional differences between *P. lobata* and its varieties can be explained by variations in the abundance of amino acids, nucleotides, saccharides, and lipids; differences in flavonoids, isoflavones, phenolic acids, organic acids, and coumarins contents caused the differences in the medicinal quality of *P. lobata* and its varieties. Additionally, the key metabolites responsible for the classification of the three *Pueraria* varieties were identified. This study provides new insights into the underlying metabolic causes of nutritional and medicinal variation in *P. lobata* and its varieties.

## 1. Introduction

The plants of the Leguminosae family are usually known as sources of starch, protein, and oil, with high economic value [1,2]. Additional species within this family can serve as medicinal plants due to their bioactive ingredients [3,4,5]. *Pueraria* is a typical representative genus comprising more than 20 species, all of which are native to Southern, Eastern, and Southeast Asia. *P. lobata* and *P*. *lobata* var. thomsonii are well-known edible and medicinal plants: *P. lobata,* i.e., kudzu, is an original variety that has a high isoflavone content with potential medicinal value and its starch has been used for curing and eating; compared with *P.* *lobata*, *P*. *lobata* var. thomsonii is a variety that shows higher starch content, and thus is called starch kudzu [6]. According to the Chinese Pharmacopoeia, the dried roots of *P. lobata* and *P*. *lobata* var. thomsonii have been used as traditional Chinese medicine for the treatment of fever, hypolipidemic, cerebrovascular, and alcohol abuse problems in China for thousands of years [7,8,9,10]. In addition, *P*. *lobata* var. montana is another variety that is notorious as an invasive species after being introduced from Asia to the United States in the 1800s, originally for fodder and soil erosion control [11,12,13].

The nutritional quality of plants mainly depends on their primary metabolites, such as amino acids, nucleotides, polysaccharides, lipids, and vitamins, whereas secondary metabolites contain physiologically active substances that can have a great impact on human health [14,15,16]. All species in the *Pueraria* genus contain different contents of bioactive flavonoid and isoflavone, including kaempferol, acacetin, puerarin, daidzein, and genistein, which have radical scavenging functions and exhibit many pharmacological properties [17,18,19]. In recent years, the research on *Pueraria* has mainly focused on the differences in starch, mineral elements, and some specific secondary metabolites such as puerarin [10,20]. However, most of these studies focused on specific classes of metabolites, therefore, can only explain the differences from one perspective. With the development of metabolomics and its successful application in many plants [21], it provides a good opportunity to understand the metabolite differences among *P. lobata* and its two varieties that affect its nutritional and medicinal quality, especially in *P. lobata* var. montana, an invasive species.

To better understand the nutritional and medicinal variations of *P. lobata* and its two varieties, ultra performance liquid chromatography-tandem mass spectrometry (UPLC-MS/MS)-based widely targeted metabolome analysis was performed. We mainly analyzed the composition and content of the nutritional and medicinal elements among *P. lobata* and its two varieties The results revealed the basic material basis of nutritional and medicinal value in *Pueraria*, and provided new evidence explaining why *P. lobata* and *P. lobata* var. thomsonii can be used as edible and medicinal plants, whereas *P. lobata* var. montana cannot.

## 2. Results

### 2.1. Widely Targeted Metabolome Analysis in P. lobata and Its Two Varieties

**Figure 1:**
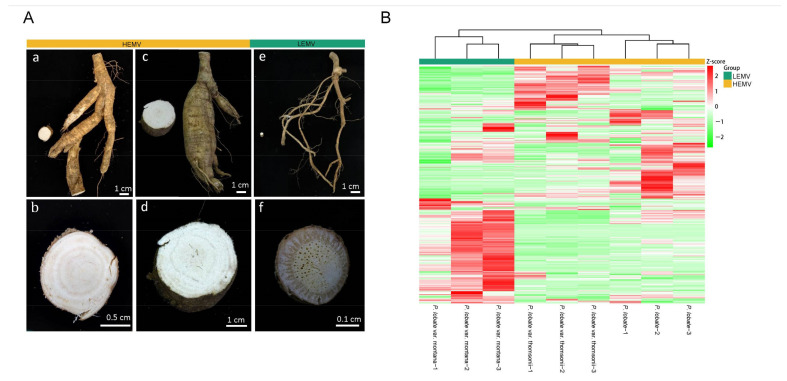
Phenotype of *P. lobata* and its two varieties. (**A**) One-year-old root tuber of *P. lobata* (**a**,**b**), *P. lobata* var. thomsonii (**c**,**d**), and *P. lobata* var. montana (**e**,**f**). Scale bar = 1 cm. (**B**) Cluster analysis of metabolites from root tuber samples of *P. lobata*, *P. lobata* var. thomsonii, and *P. lobata* var. montana. The color indicates the level of accumulation of each metabolite, from low (green) to high (red). The Z-score represents the deviation from the mean by standard deviation units.

According to the nutritional and medicinal quality, *P. lobata* and its variety *P. lobata* var. thomsonii belong to the high nutritional and medicinal value (HNMV) group and *P. lobata* var. *montana* belongs to the low nutritional and medicinal value (LNMV) group. To better understand the nutritional and medicinal differences among *Pueraria* species, we performed widely targeted UPLC-MS/MS-based metabolite profiling of *P. lobata*, *P. lobata* var. thomsonii, and *P. lobata* var. montana (Figure 1A). In total, 614 metabolites were identified, of which there were a large number of primary metabolites likely to contribute to the nutritional quality, as well as secondary metabolites likely to contribute to the medicinal quality (Figure 1B, Table S1).

The comparison among *P. lobata*, *P. lobata* var. thomsonii, and *P. lobata* var. montana showed that *P. lobata* has higher amounts of syringaresinol-4’-O-glucoside and disinapoyl glucoside; *P. lobata* var. thomsonii has higher amounts of glycycoumarin and 2-hydroxyadenosine; and *P. lobata* var. montana has higher amounts of p-coumaroylputrescine, tricin-7-O-(6″-malonyl)-glucoside, and glycitin. These results suggest that the specific compounds found in a particular variety can be used to differentiate the *Pueraria* varieties.

### 2.2. Multivariate Analysis of Identified Metabolites

**Figure 2:**
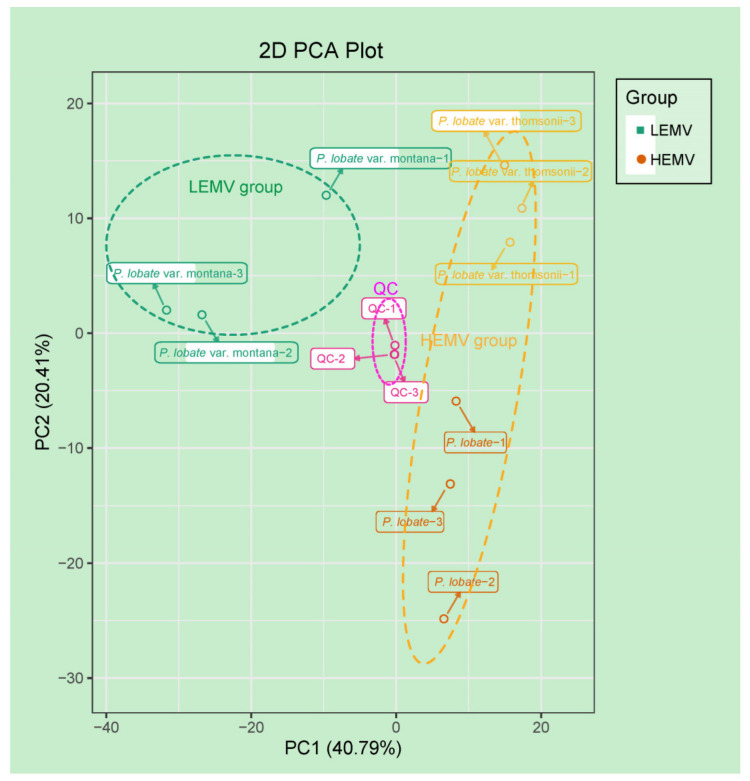
Differential root tuber metabolites among *P. lobata*, *P. lobata* var. thomsonii, and *P. lobata* var. montana. PCA of metabolites identified from *P. lobata*, *P. lobata* var. thomsonii, and *P. lobata* var. montana. Equal volumes of *P. lobata*, *P. lobata* var. thomsonii, and *P. lobata* var. montana root tuber samples were mixed for use as a quality control (QC).

To examine all the metabolomic differences among the three *Pueraria* varieties and the variability between the HNMV and LNMV groups, the data set from the 614 metabolites was subjected to principle component analysis (PCA). PCA clearly separated the three *Pueraria* varieties and quality control (QC) samples with a significance of 0.01, and the repeated samples were tightly gathered, indicating the repeatability and reliability of the experiments. The PCA showed a total of 61.20% model variance described by the PCA 1 and PCA 2 axes, which revealed significant metabolomic differences according to the PCA 1 axis among the three *Pueraria* varieties (Figure 2). This analysis revealed three distinct groups associated with distinct metabolite profiles of the three *Pueraria* varieties.

**Figure 3:**
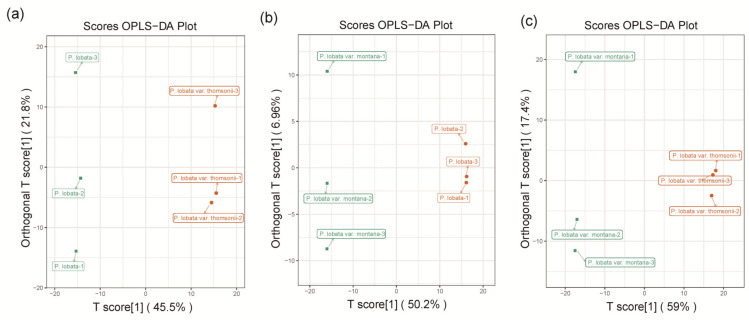
OPLS-DA of metabolites identified among *P. lobata*, *P. lobata* var. thomsonii, and *P. lobata* var. montana (**a**–**c**). OPLS-DA model plots for the comparison group *P. lobata* versus *P. lobata* var. thomsonii, *P. lobata* versus *P. lobata* var. montana, and *P. lobata* var. thomsonii versus *P. lobata* var. montana, respectively.

To further determine the differences in the metabolite composition between intra-group samples, supervised orthogonal signal correction was applied and a partial least squares-discriminant analysis (OPLS-DA) model was used to amplify the differences between different groups (Figure 3). In this study, the OPLS-DA model compared the metabolite content of the samples in pairs to evaluate the difference between *P. lobata* and *P. lobata* var. thomsonii (R2X = 0.455, R2Y = 0.972, Q2 = 0.906), *P. lobata* and *P. lobata* var. montana (R2X = 0.502, R2Y = 1, Q2 = 0.915), and *P. lobata* var. thomsonii and *P. lobata* var. montana (R2X = 0.590, R2Y = 994, Q2 = 0.940) (Figure 3). The Q2 values of all the comparison groups exceeded 0.9, thus demonstrating that these models were stable and reliable and could be used to further screen for differential metabolites.

Then, according to *t*-test *p*-values being all less than 0.05 and variable importance in projection (VIP) values being ≥1.0) from the OPLS-DA model, 127 significantly different metabolites were identified, of which 83 metabolites were higher and 44 metabolites were lower in the HNMV group than in the LNMV group. The 127 metabolites were categorized into 11 different classes (Table S2), of which amino acids, nucleotides, saccharides, phenolic acids, flavonoids, organic acids, and coumarins accounted for the vast majority (Figure S1).

Subsequently, we conducted KEGG pathway enrichment analysis to identify differences in the metabolic pathways between the HNMV and LNMV groups. KEGG classification of differential metabolites showed that the differential metabolites between the HNMV and LNMV groups were significantly enriched in the biosynthesis pathway of isoflavonoid, phenylpropanoid, folate, and arginine (Figure S2).

### 2.3. Nutritional and Medicinal Component Differences among P. lobata and Its Two Varieties

**Figure 4:**
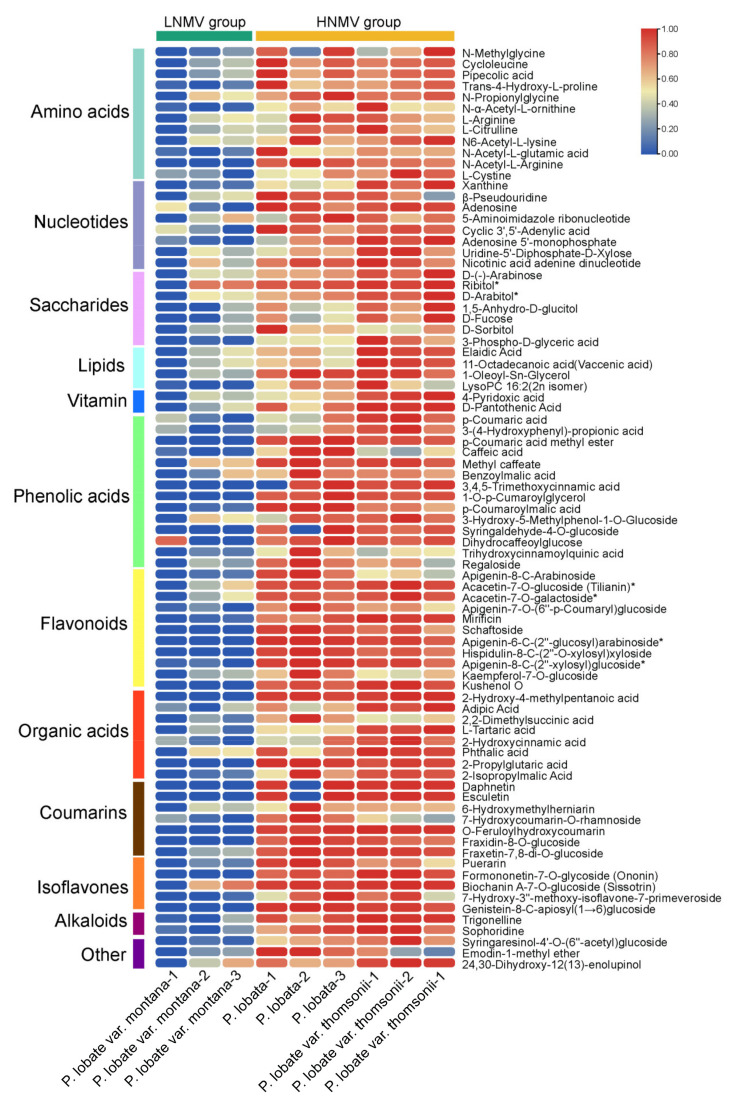
Heatmap of metabolites from root tuber samples of the HNMV group and the LNMV group. The color indicates the level of accumulation of each metabolite, from low (green) to high (red).

We focused on classes of metabolites likely to be major contributors to the nutritional and medicinal qualities among *P. lobata* and its two varieties. Based on Log_2_FC (fold change) and VIP values, we screened out 83 upregulated substances between the HNMV group and the LNMV group (Figure 4, Table S3). In detail, 33 primary metabolites comprising 12 amino acids, 8 nucleotides, 7 saccharides, 4 lipids, and 2 vitamins were identified. Of these, the majority of nucleotides and saccharides were significantly greater in the HNMV group than in the LNMV group (Log_2_FC ≥ 3), whereas the majority of amino acids, lipids, and vitamins exhibited differences (1 ≤ Log_2_FC ≤ 3), suggesting that these primary metabolites are involved in the nutritional differences between the HNMV group and the LNMV group. Furthermore, 50 secondary metabolites comprising 14 phenolic acids, 11 flavonoids, 8 organic acids, 7 coumarins, 5 isoflavones, 2 alkaloids, 1 lignan, 1 quinone, and 1 terpenoid were identified. Of these, the majority of flavonoids, isoflavones, phenolic acids, coumarins, and alkaloids was significantly greater in the HNMV group than in the LNMV group (Log_2_FC ≥ 3), whereas the majority of organic acids and lignan exhibited differences (1 ≤ Log_2_FC ≤ 3). Moreover, most of the identified the flavonoids and isoflavones were glycosyl derivatives of apigenin, acacetin, kaempferol, puerarin, formononetin, and genistein.

**Figure 5:**
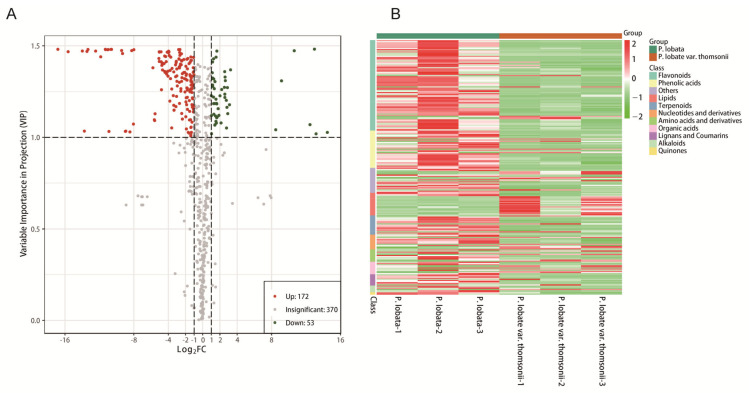
Differentially accumulating metabolites between *P. lobata* and *P. lobata* var. thomsonii. (**A**) Volcano plot of the 595 metabolites identified. Differential metabolites were defined as metabolites with a fold change of ≥2 or ≤0.5 in *P. lobata* compared to *P. lobata* var. thomsonii. A threshold of VIP ≥ 1.0 was used to separate differential metabolites from unchanged metabolites. (**B**) Cluster analysis of the differential metabolites identified between *P. lobata and P. lobata* var. thomsonii.

Although *P. lobata* and *P. lobata* var. thomsonii were both recorded in the Chinese Pharmacopoeia as having high medicinal values, modern studies have confirmed that there are considerable differences between the two varieties in terms of the material basis and efficacy [22,23,24]. To clarify the differences in the material basis between *P. lobata* and *P. lobata* var. thomsonii, 225 metabolites were screened out as significantly different in *P. lobata* and *P. lobata* var. thomsonii, of which 172 metabolites were higher and 53 metabolites were lower in *P. lobata* than *P. lobata* var. thomsonii, and the 225 different substances were mainly secondary metabolites (Figure 5A, Table S4). These results indicate that there is little resistivity difference in the nutritional value between *P. lobata* and *P. lobata* var. thomsonii. In addition, 122 upregulated secondary metabolites comprising 35 flavonoids, 28 isoflavones, 27 phenolic acids, 14 terpenoids, 8 organic acids, 6 coumarins, and 4 alkaloids and 32 downregulated secondary metabolites comprising 12 flavonoids, 5 isoflavones, 6 phenolic acids, 3 terpenoids, 3 organic acids, 1 coumarins. and 2 alkaloids were identified (Figure 5B, Table S4). In detail, most of the upregulated flavonoids and isoflavones were apigenin, kaempferol, naringenin, daidzin, formononetin, genistein, prunetin, and their glycosyl and methyl derivatives, and most of the upregulated terpenoids were soyasaponins. These secondary metabolites may be the material basis for the difference in efficacy between the two varieties.

## 3. Discussion

*Pueraria* plants are widely distributed in China, Thailand, Japan, India, and other countries in Asia. At present, only two varieties of *Pueraria* have been used for traditional Chinese medicine and food purposes. The root tubers of *P. lobata* show higher isoflavone content, especially puerarin, being an index component called kudzu in the Chinese Pharmacopoeia, whereas the root tubers of *P. lobata* var. thomsonii show higher starch content but lower isoflavone content, and thus is called starch kudzu. The tuberous roots of *P. lobata* var. montana, a variant of *Pueraria*, has little nutritional and medicinal value, however, it has the characteristics of rapid growth and strong cold tolerance, thus is regarded as a species that can cause ecological disaster [25]. Over-arching differences in the metabolic profiles of *Pueraria* plants have not been investigated thoroughly until now. In this study, we used UPLC-MS/MS-based widely targeted metabolomics to understand nutritional and medicinal variations in three representative species in *Pueraria* with different use purposes. Thus, this study provides novel evidence of metabolic causes underlying the nutritional and medicinal use differences in *Pueraria* plants.

The primary metabolites of plants form the basic nutrients necessary for human body, such as proteins, fats, carbohydrates, minerals, vitamins, and dietary fibers; for plants, these primary metabolites are necessary to maintain their life activities, growth, and development. In this study, there were significant differences in the contents of primary metabolites. In detail, the contents of D-arabinose, D-fucose, D-sorbitol and D-pantothenic acid were significantly higher in the HNMV group than in the LNMV group. According to previous studies, D-arabinose, D-fucose, and D-sorbitol have a refreshing sweet taste, which may partially explain the sweeter taste of *P. lobata* and *P. lobata* var. thomsonii. Moreover, D-pantothenic acid, also known as vitamin B5, can act on normal epithelial organs such as nerves, adrenal glands, the digestive tract, and skin to improve the resistance of animals to pathogens. Furthermore, vitamin B5 can also increase glutathione biosynthesis, and thus slow down apoptosis and damage. To some extent, these finding reveal the reason why *P. lobata* and *P. lobata* var. thomsonii can be used as food, unlike *P. lobata* var. montana. In addition, the upregulated primary metabolites between *P. lobata* and *P. lobata* var. thomsonii were amino acids, nucleotides, and saccharides, whereas the downregulated primary metabolites were lipids, indicating that the nutritional values of *P. lobata* and *P. lobata* var. thomsonii are significantly different.

Secondary metabolites can produce the quality, flavor and immune function substances of agricultural products, which have medicinal value or serve the function of promoting human health [26,27]. Our widely targeted metabolite profiling analysis result identified 127 differential metabolites in three representative species of *Pueraria*. Comparing the secondary metabolites of *P. lobata*, *P. lobata* var. thomsonii, and *P. lobata* var. montana, 50 secondary metabolites were higher in the HNMV group than in the LNMV group. Most of them were flavonoids, isoflavones, and phenolic acids, followed by organic acids and coumarins, and a very small number of alkaloids and lignans. Previous studies showed that isoflavones are exclusively accumulated in legumes, such as soybeans, and play an important role in plant defense and nodules [28]. In this study, 15 flavonoids and 8 isoflavones were highly accumulated in the HNMV group compared with the LNMV group. Most of them were puerarin, daidzin, genistein, formononetin, acacetin, apigenin, and their derivatives, which were modified by glycosylation or methylation, and these modified metabolites greatly affect the medicinal value of isoflavone products. Thus, our results suggest that differences in both the composition and abundance of flavonoids and isoflavones may contribute to the medicinal use differences.

Although *P. lobata* and *P. lobata* var. thomsonii are both recorded in the Chinese Pharmacopoeia, the content of flavonoids, isoflavones, and terpenoids showed significant differences, implying that their pharmacological effects are also different, which is worthy of further study. Interestingly, the content of soyasaponins showed significant differences between *P. lobata* and *P. lobata* var. thomsonii, but showed similarities between the HNMV group and the LNMV group. In addition, *P. lobata* var. montana contained a higher content of some flavonoids, phenolic acids, and alkaloids such as glycitin, naringenin-7-O-(6″-malonyl)-glucoside, apigenin-7-O-(6″-malonyl)-glucoside, N-feruloylputrescine, glucosyringic acid, and 3,4,5-trimethoxyphenyl-1-O-glucoside, which possess good anti-inflammatory activity [29], suggesting that *P. lobata* var. montana has the potential to be developed and utilized for its medicinal value.

UPLC-MS/MS-based widely targeted metabolome analysis is also a useful tool to differentiate varieties [30]. In this study, the PCA applied to the chemical composition found in the root tubers of *Pueraria lobata* and its varieties allowed a high degree of *Pueraria* variety differentiation according to metabolomic differences. In detail, p-coumaroylputrescine, tricin-7-O-(6″-malonyl)-glucoside, and glycitin were higher in *P. lobata* var. montana than in *P. lobata* and *P. lobata* var. thomsonii; glycycoumarin and 2-hydroxyadenosine were higher in *P. lobata* var. thomsonii than in *P. lobata* and *P. lobata* var. montana; syringaresinol-4′-O-glucoside and disinapoyl glucoside were higher in *P. lobata* than in *P. lobata* var. thomsonii and *P. lobata* var. montana. These discriminating metabolites could represent useful quality markers to differentiate *P. lobata*, *P. lobata* var. thomsonii, and *P. lobata* var. montana.

## 4. Materials and Methods

### 4.1. Sample Preparation and Extraction

As traditional Chinese medicine, the dry root tubers of *P. lobata* and *P. lobata* var. thomsonii are recorded in the Chinese Pharmacopoeia. One-year-old root tubers were collected from one plant to create one biological sample. Three biological samples were created for each *Pueraria* variety. Samples were stored at −80 °C until metabolite extraction. The lyophilized sample was crushed using a mixer mill (MM 400, Retsch) with a zirconia bead for 1.5 min at 30 Hz. A total of 100 mg powder was weighed and extracted overnight at 4 °C with 1.2 mL 70% aqueous methanol. Following centrifugation at 10,000× *g* for 10 min, the extracts were filtrated using 0.22 μm membrane (SCAA-104, ANPEL, Shanghai, China, http://www.anpel.com.cn/ (accessed on 25 October 2021)) before UPLC-MS/MS analysis. The quality control sample (QC) was prepared by mixing all the samples and used to demonstrate the precision of the assay. During the instrumental analysis, a quality control sample was inserted into each of the three test samples to examine the repeatability of the analysis process.

### 4.2. UPLC Conditions

Root tuber extracts were analyzed using a UPLC-ESI-MS/MS system (UPLC, SHIMADZU Nexera X2, www.shimadzu.com.cn/ (accessed on 25 October 2021); MS, Applied Biosystems 4500 Q TRAP, www.appliedbiosystems.com.cn/ (accessed on 25 October 2021)). The analytical conditions were as follows: UPLC: column, Agilent SB-C18 (1.8 μm, 2.1 mm × 100 mm); the mobile phase consisted of solvent A, pure water with 0.1% formic acid; and solvent B, acetonitrile with 0.1% formic acid. Sample measurements were performed using a gradient program that employed the starting conditions of 95% A, and 5% B. Within 9 min, a linear gradient to 5% A, 95% B was programmed, and a composition of 5% A, 95% B was kept for 1 min. Subsequently, a composition of 95% A, 5.0% B was adjusted within 1.10 min and maintained for 2.9 min. The column oven was set to 40 °C; the injection volume was 4 μL. The effluent was alternatively connected to an ESI-triple quadrupole-linear ion trap (QTRAP)-MS.

### 4.3. ESI-Q TRAP-MS/MS

Linear ion trap (LIT) and triple quadrupole (QQQ) scans were acquired on a triple quadrupole-linear ion trap mass spectrometer (Q TRAP), AB4500 Q TRAP UPLC/MS/MS system, equipped with an ESI Turbo Ion-Spray interface, operating in positive and negative ion mode and controlled by Analyst 1.6.3 software (AB Sciex, Singapore). The ESI source operation parameters were as follows: ion source, turbo spray; source temperature 550 °C; ion spray voltage (IS) 5500 V (positive ion mode)/−4500 V (negative ion mode); ion source gas I (GSI), gas II(GSII), and curtain gas (CUR) were set to 50, 60, and 25.0 psi, respectively; the collision gas (CAD) was high. Instrument tuning and mass calibration were performed with 10 and 100 μmol/L polypropylene glycol solutions in QQQ and LIT modes, respectively. QQQ scans were acquired as multiple reaction monitoring (MRM) experiments with the collision gas (nitrogen) set to medium. To produce maximal signals, collision energy (CE) and declustering potential (DP) were optimized for individual MRM transitions. A specific set of MRM transitions was monitored for each period according to the metabolites eluted within the period. The chromatogram for each elution period is provided in Figure S3.

### 4.4. Qualitative and Quantitative Analysis of Metabolites

Mass spectrometry (MS) data acquisition and processing were performed as described previously [31]. Based on the Metware in-house MS^2^ spectral tag (MS2T) library (Metware Biotechnology Co., Ltd., Wuhan, China), the metabolites of the samples were qualitatively and quantitatively analyzed by mass spectrometry. The characteristic ions of each substance were screened out by the triple quadrupole rod, and the signal strength of the characteristic ions was obtained in the detector. The mass spectrometry file under the sample was opened with MultiaQuant software 3.0.3 to carry out the integration and correction of the chromatographic peaks, and the relative content of the corresponding substances in the peak area of each chromatographic peak were calculated. Finally, all chromatographic peak area integral data were derived. To compare the contents of each metabolite in different samples, we calibrated the mass spectrum peaks detected by each metabolite in different samples based on the information of metabolite retention time and peak pattern. Thus, the accuracy of the qualitative and quantitative analysis was further ensured. The retention time data of 30 representative metabolites are provided in Table S5.

### 4.5. Statistical Analysis

Unsupervised principle component analysis (PCA) was carried out using the statistics function prcomp within R v3.5.0 (www.r-project.org (accessed on 25 October 2021)). Supervised multiple regression orthogonal partial least-squares discriminant analysis (OPLS-DA) was performed using ropls v1.19.8 in R.

### 4.6. Hierarchical Cluster Analysis and Pearson Correlation Coefficients

The hierarchical cluster analysis (HCA) results of the samples and the metabolites are presented as heatmaps with dendrograms, while Pearson correlation coefficients (PCCs) between samples were calculated by the cor function in R and are presented as only heatmaps. Both HCA and PCC were carried out by the R package pheatmap. For HCA, normalized signal intensities of metabolites (unit variance scaling) are visualized as a color spectrum.

### 4.7. Differential Metabolites Analysis

Significantly regulated metabolites between groups were determined by variable importance in projection (VIP) ≥ 1 and absolute Log_2_FC (fold change) ≥ 1. VIP values were extracted from the OPLS-DA results, which also contained score plots and permutation plots, generated using R package MetaboAnalystR. The data were log-transformed (log2) and mean-centered before OPLS-DA. To avoid overfitting, a permutation test (200 permutations) was performed.

### 4.8. KEGG Annotation and Enrichment Analysis

Identified metabolites were annotated using the KEGG compound database (http://www.kegg.jp/kegg/compound/ (accessed on 25 October 2021)), annotated metabolites were then mapped to the KEGG pathway database (http://www.kegg.jp/kegg/pathway.html (accessed on 25 October 2021)). Pathways in which significantly regulated metabolites were mapped were then fed into metabolite sets enrichment analysis (MSEA) and their significance was determined by the hypergeometric test’s *p*-values.

## 5. Conclusions

In summary, we successfully performed UPLC-MS/MS-based metabolic analysis to systematically compare nutritional and medicinal differences among *P. lobata*, *P. lobata* var. thomsonii, and *P. lobata* var. montana. This work provides comprehensive information on both metabolite compositions and abundances in *Pueraria*. The results infer that the differences in primary metabolites including amino acids, nucleotides, and saccharides affect the nutritional value of the three *Pueraria* varieties. On the other hand, the differences in the content of puerarin, daidzin, genistein, formononetin, acacetin, apigenin and their derivatives are the main factors affecting the medicinal quality of *P. lobata*, *P. lobata* var. thomsonii, and *P. lobata* var. montana. In addition, phenolic acids, organic acids, and coumarins constitute the material basis of the three *Pueraria* varieties’ medicinal quality. The key metabolites responsible for the classification of three *Pueraria* varieties are p-coumaroylputrescine, tricin-7-O-(6″-malonyl)-glucoside, glycitin, glycycoumarin, 2-hydroxyadenosine, syringaresinol-4′-O-glucoside, and disinapoyl glucoside.

## Data Availability

Not applicable.

## Supplementary Materials

Figure S1: Differentially accumulating metabolites between HNMV and LNMV group, Figure S2: Metabolic pathways enrichment analysis for HNMV and LNMV group differentially accumulated metabolites, Figure S3: Total ion chromatogram (TIC) of each samples, Table S1: Widely targeted UPLC-MS/MS based metabolite profiling of P. lobata, P. lobata var. thomsonii and P. lobata var. montana, Table S2: Statistics of differentially metabolites in the root tuber between HNMV group and LNMV group, Table S3: Statistics of up-regulated metabolites in the root tuber between HNMV group and LNMV group, Table S4: Statistics of differentially metabolites in the root tuber between P. lobata and P. lobata var. thomsonii, Table S5: The retention time data of 30 representative metabolites.
