# Supplementary material for: Identification of Nutritional Ingredients and Medicinal Components of Pueraria lobata and Its Varieties Using UPLC-MS/MS-Based Metabolomics
Source: Molecules. 2021 Oct 30;26(21):6587. doi: 10.3390/molecules26216587 (PMC8588241; doi:10.3390/molecules26216587)
Supplement: Supplementary file 1 [file molecules-26-06587-s001.zip › spp Figure.pdf]

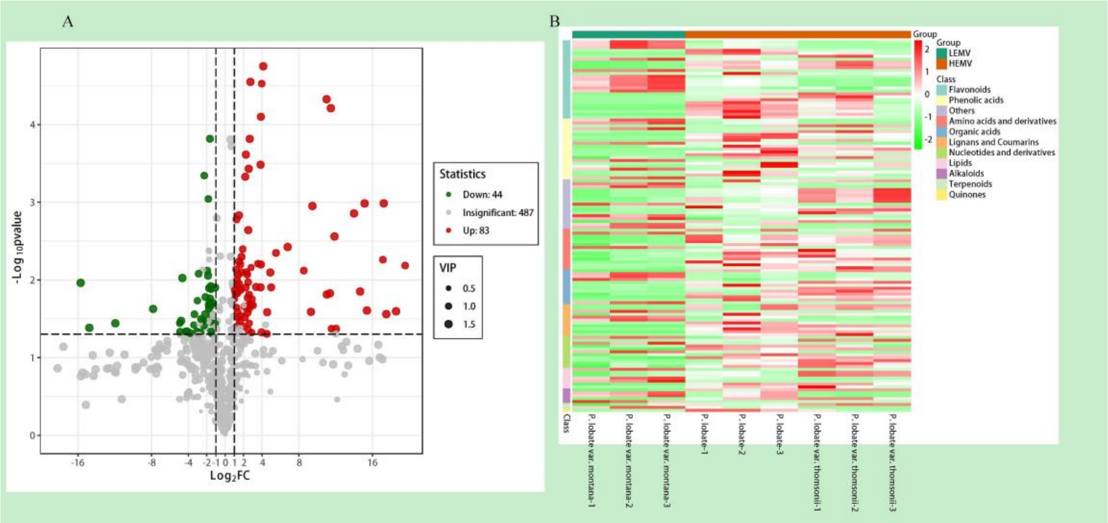

Figure S1. Differentially accumulating metabolites between HNMV and LNMV group. (**A**) Volcano plot of the 614 metabolites identified. Differential metabolites were defined as metabolites with fold change ≥ 2 or ≤ 0.5 in HNMV group compared to LNMV group. A threshold of VIP ≥ 1.0 was used to separate differential metabolites from unchanged metabolites. (**B**) Cluster analysis of the differential metabolites identified between HNMV and LNMV group.

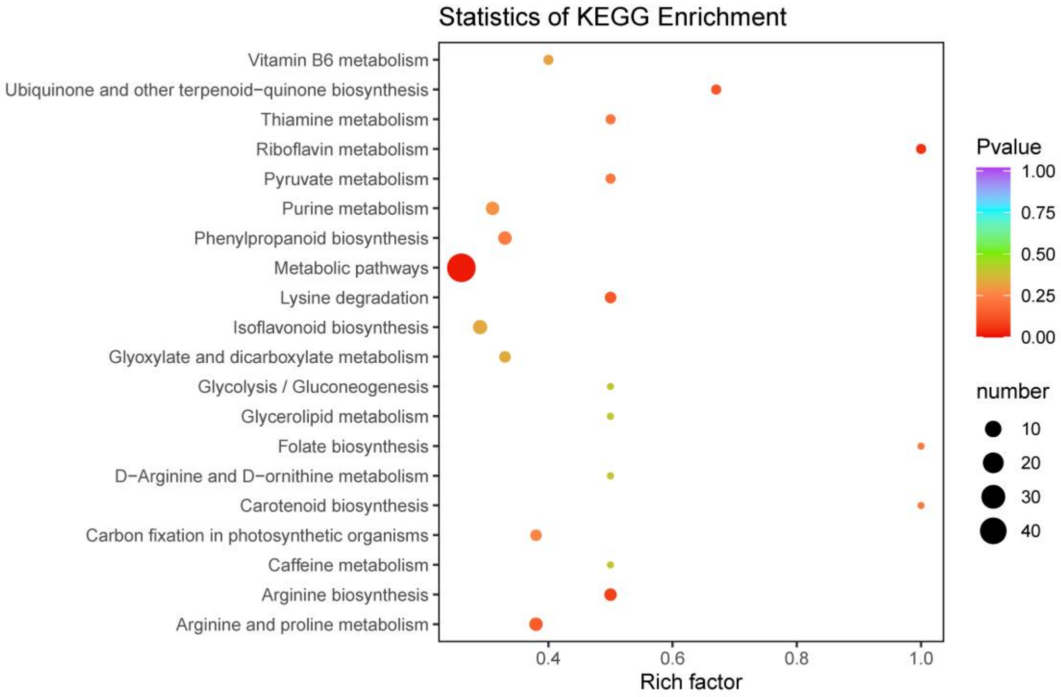

Figure S2. Metabolic pathways enrichment analysis for HNMV and LNMV group differentially accumulated metabolites.

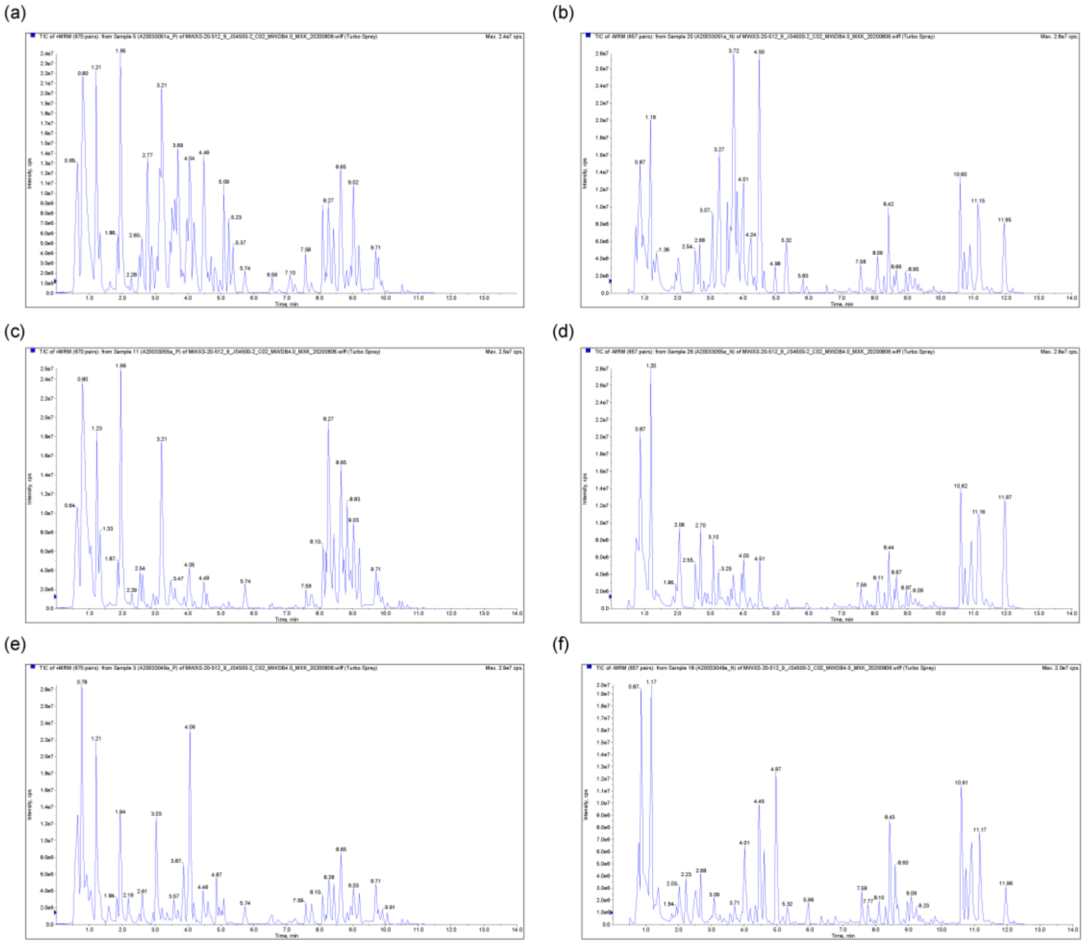

Figure S3. Total ion chromatogram (TIC) of each samples. TIC of *P. lobata* under positive ion mode (**a**) and negative ion mode (**b**); TIC of *P. lobata* var. thomsonii under positive ion mode (**c**) and negative ion mode (**d**); TIC of *P. lobata* var. montana under positive ion mode (**e**) and negative ion mode (**f**).
